# Evolution of surgery in advanced epithelial ovarian cancer in a dedicated gynaecologic oncology unit—seven year audit from a tertiary care centre in a developing country

**DOI:** 10.3332/ecancer.2014.422

**Published:** 2014-04-17

**Authors:** Anupama Rajanbabu, Santhosh Kuriakose, Sheikh Zahoor Ahmad, Tejal Khadakban, Dhiraj Khadakban, R Venkatesan, D K Vijaykumar

**Affiliations:** Department of Surgical and Gynaecologic Oncology, Amrita Institute of Medical Sciences and Amrita Vishwavidyapeetham, Kochi, Kerala, India

**Keywords:** advanced ovarian cancer, primary cytoreduction, interval cytoreduction

## Abstract

**Aims:**

1. To audit our performance as a dedicated gynaecologic oncology unit and to analyse how it has evolved over the years.
2. To retrospectively evaluate the outcome of advanced ovarian cancer treated with neoadjuvant chemotherapy (NACT) followed by interval surgery versus upfront surgery.

**Methods and results:**

One hundred and ninety-eight patients with advanced epithelial ovarian cancer (EOC) who were treated from 2004 to 2010 were analysed. Eighty-two patients (41.4%) underwent primary surgery and 116 (58.6%) received NACT. Overall, an optimal debulking rate of 81% was achieved with 70% for primary surgery and 88% following NACT. The optimal cytoreduction rate has improved from 55% in 2004 to 97% in 2010. In primary surgery, the optimal debulking rate increased from 42.8% in 2004 to 93% in 2010, whereas in NACT group the optimal cytoreduction rate increased from 60% to 100% by 2010.

On the basis of the surgical complexity scoring system it was found that surgeries with intermediate complexity score had progressively increased over the years.

There was a mean follow-up of 21 months ranging from 6 to 70 months. The progression-free survival and overall survival (OS) in patients undergoing primary surgery were 23 and 40 months, respectively, while it was 22 and 40 months in patients who received NACT. However, patients who had suboptimal debulking, irrespective of primary treatment, had significantly worse OS (26 versus 47 months) compared with those who had optimal debulking.

**Conclusions:**

As a dedicated gynaecologic oncology unit there has been an increase in the optimal cytoreduction rates. The number of complex surgeries, as denoted by the category of intermediate complexity score, has increased.

Patients with advanced EOC treated with NACT followed by interval debulking have comparable survival to the patients undergoing primary surgery. Optimal cytoreduction irrespective of primary modality of treatment gives better survival.

## Background

Major shifts have occurred in the management of EOC over the last few years. Even though primary cytoreductive surgery has been the standard clinical practice [1–3] this has been challenged by many studies [4–6]. The definition of optimal cytoreductive surgery has undergone repeated revisions [3, 7, 8]. The aim of this study was to audit our performance and to see whether our surgical expertise has improved by concentrating on gynaecologic oncology as a separate specialty.

## Methods

We included consecutive cases of advanced ovarian cancers (Stage IIIB and above) operated on from 1st January 2004 to 31st December 2010 at the Amrita Institute of Medical Sciences, Kerala, India. Approval was obtained from the institutional review board and data were retrieved from the patients’ records and the institutional tumour registry. There were 198 cases and their clinical records were reviewed. Optimal debulking was defined as residual tumour less than 1 cm since the data analysed are before 2010. Before 2010 residual tumour less than 1 cm was considered as optimal cytoreduction [9]. Data were analysed with SPSS (V.17.0).

## Results

One hundred ninety-eight patients with advanced ovarian cancer were operated on at our institute during this period. Patient characteristics are given in Table 1.

Preoperatively, patients were evaluated by tumour markers, CT scan, or MRI scan after a clinical examination. All cases were discussed by the multidisciplinary tumour board before initiating treatment. The decision between primary surgery and neoadjuvant chemotherapy (NACT) was taken based on age, performance status, and clinical impression of operability and CT scan or MRI assessment. An age of less than 80, ECOG –PS 2, or above, liver or lung parenchymal metastasis, metastasis at porta hepatis and mesenteric root, extensive peritoneal carcinomatosis were considered to be more in favour of NACT. Factors like ascites, CA 125, and primary tumour burden, either alone or in combination were not taken as criteria for NACT. Diagnostic laparoscopy was used in cases where operability was doubtful. Twelve patients had diagnostic laparoscopy during this period to assess operability; six went on to have primary surgery and achieved optimal debulking. The remaining six patients had extensive peritoneal disease or disease involving small bowel mesentry, and hence only a biopsy was done and NACT was given.

## Trends in surgery

**Figure 1:**
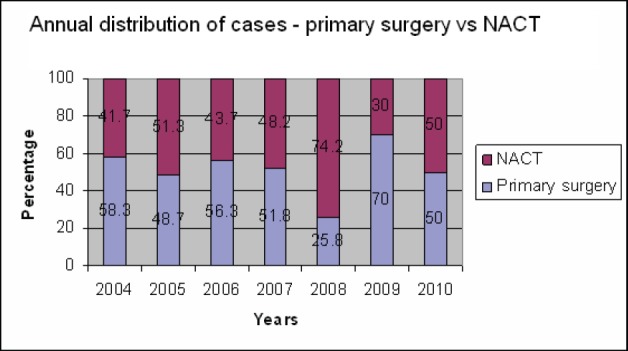
Annual trends in surgery.

**Figure 2:**
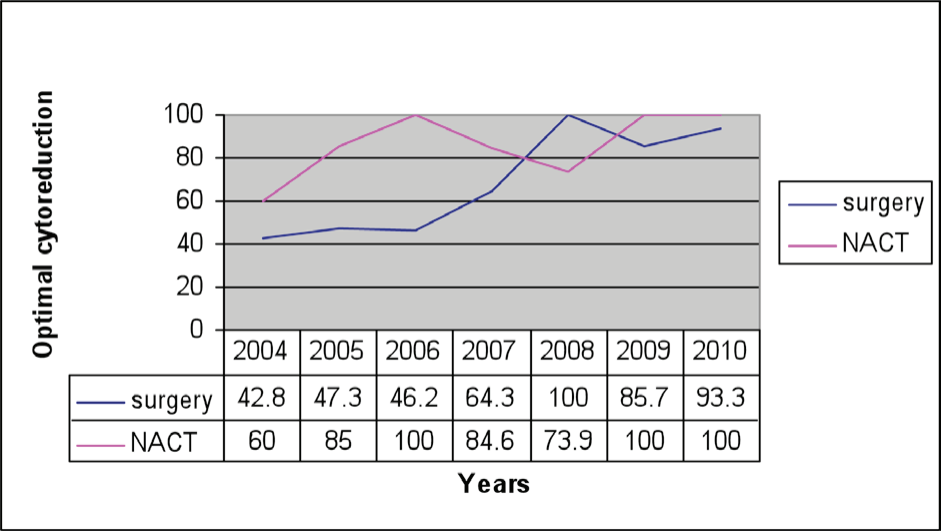
Optimal cytoreduction comparison between primary surgery and NACT.

The primary surgery rate showed an initial declining trend between 2005 and 2008, but that was reversed in the subsequent years Figure 1. Overall, an optimal debulking rate of 81% was achieved with 70% in the primary surgery group and 88% following NACT. In the primary cytoreductive surgery group, the optimal debulking rate increased from 42.8% in 2004 to 93% in 2010, whereas in the NACT group this increased from 60% to 100% by 2010 (Figure 2).

In the 38 patients who had suboptimal debulking, the sites of disease which made debulking suboptimal in the primary surgery and NACT groups are shown in Table 2. Initial laparoscopic assessment may have changed the management in 23 of these 38 patients who had extensive peritoneal disease and small bowel mesenteric disease resulting in suboptimal cytoreduction.

**Figure 3:**
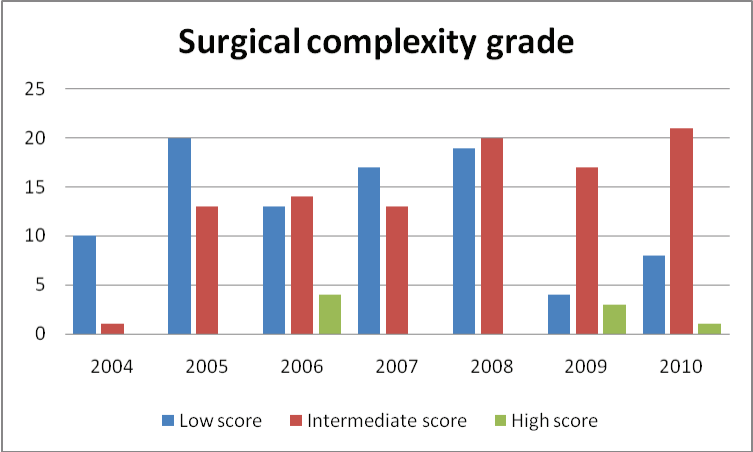
Surgical complexity grades over the years.

**Figure 4:**
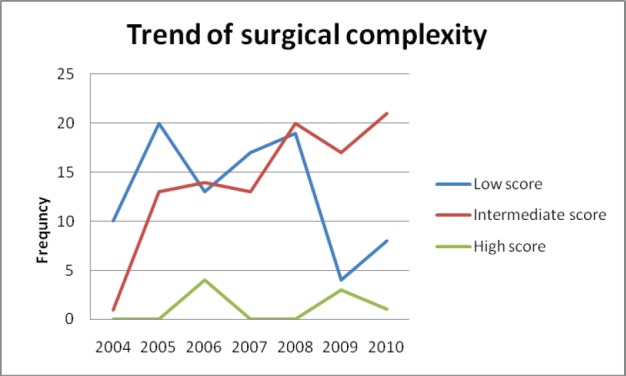
Trend of surgical complexity over the years.

The surgical complexity scoring system [10] was used to categorize the surgeries performed over the years (Figures 3 and 4).

Complicated surgical procedures like intestinal resection anastomosis, stripping of diaphragm, pelvic and para-aortic lymphadenectomy has increased over years as evidenced by the upward trend of intermediate surgical complexity score. In complicated cases requiring bowel resection or extensive dissection of urinary tract, assistance of appropriate surgical specialities (urologist and surgical gastroenterologist) was obtained during surgery. Pelvic and para-aortic lymphadenectomies were done only if nodes were found to be enlarged in imaging or during intra op evaluation.

### Complications following surgery

Comparing the complications following primary and interval debulking surgery we found that patients undergoing primary surgery had significantly longer surgery and more blood loss and also more intraoperative hypotension requiring inotropic support. There was no difference in the post operative ileus or post operative infection and intensive care unit stay but duration of hospital stay was longer in the primary surgery group.

Platinum either alone or in combination was given in 96% of cases, and 80% of cases were treated with a combination of platinum and paclitaxel.

### Follow-up

There was a mean follow-up of 21 months with a minimum of six months and maximum of 70 months. The disease status of the patients after this follow up is shown in Table 3.

### Outcome analysis based on primary modality of treatment

**Figure 5:**
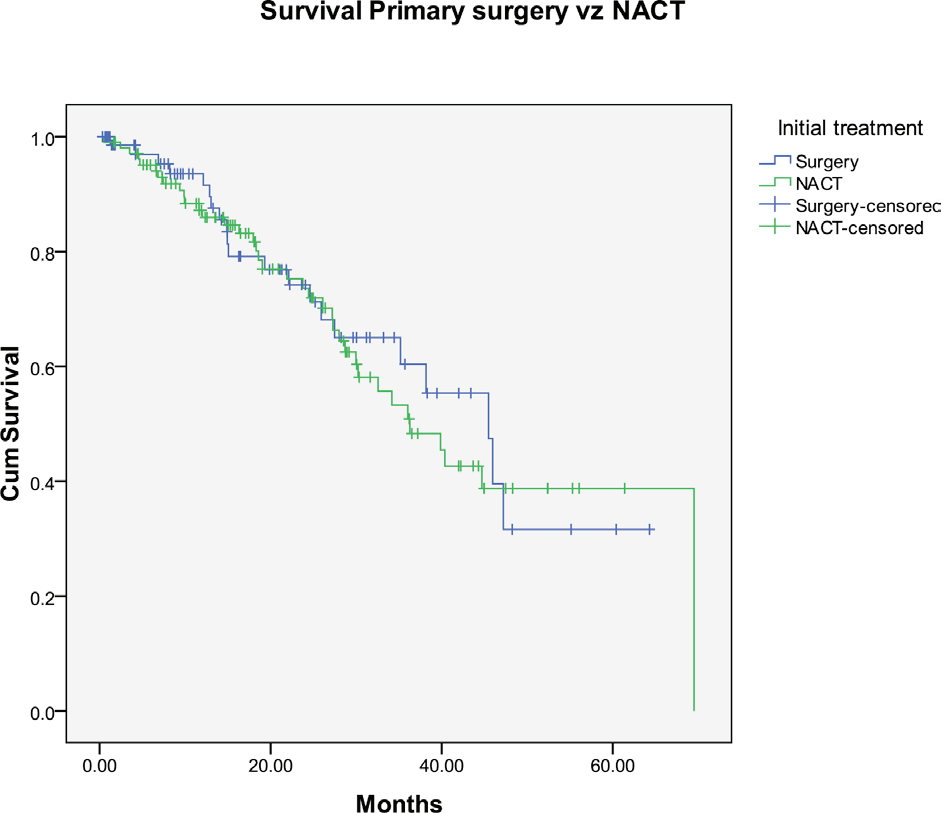
Kaplan–Meier survival graph comparing the OS—primary surgery versus NACT.

**Figure 6:**
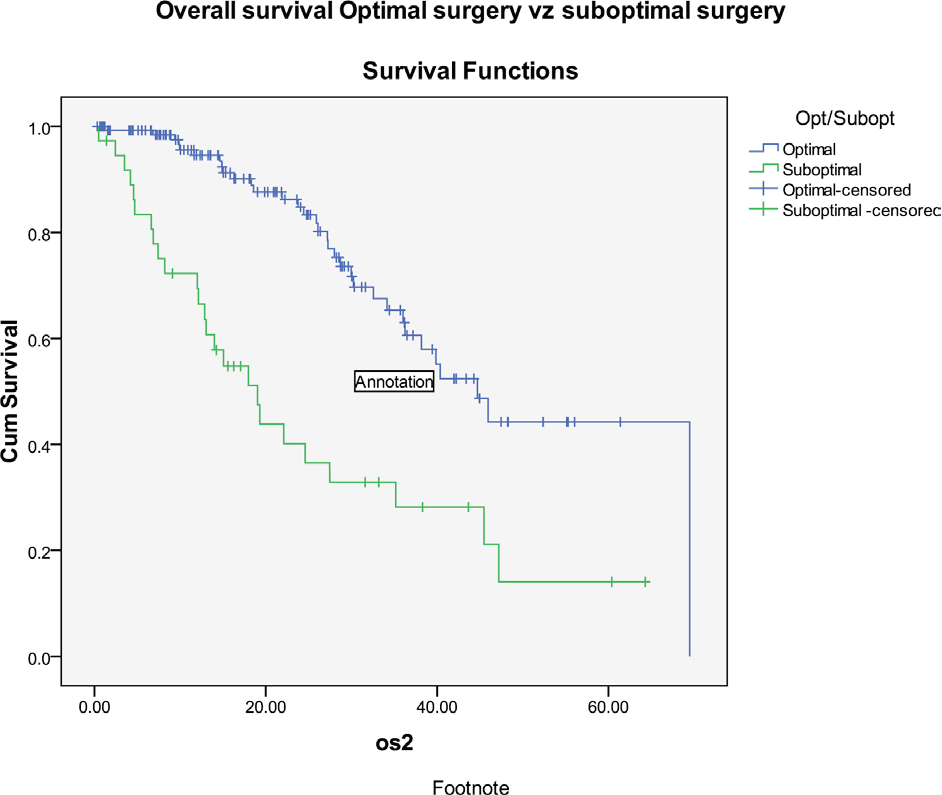
Kaplan–Meier survival graph comparing the OS—optimal versus suboptimal debulking.

The progression-free survival and overall survival (OS) in patients undergoing primary surgery were 23 and 40 months, respectively, while it was 22 and 40 months in patients who received NACT (Figure 5). Patients who had suboptimal debulking, irrespective of primary treatment, had significantly worse OS (26 versus 47 months) compared to those who had optimal debulking (Figure 6).

## Discussion

Complete resection of all macroscopic disease at primary debulking surgery has been shown to be the single most important independent prognostic factor in advanced ovarian carcinoma [3, 7, 11, 12]. The comparison of OS of patients treated by surgeons with and without subspecialist training in gynaecologic oncology showed a survival benefit for those that were treated by the former. This is because of the optimal debulking rates of 75% achieved in the hands of a trained gynaecologic oncologist [8]. The optimal cytoreduction rate, which was only 48% for primary surgery during the early stages of our career has increased to 93% by 2010. A similar trend was noted (from 60% to 100%) in interval surgery after NACT. Our surgical unit consists of a surgical oncologist with special interest in gynaec oncology, a gynaecologist trained in gynaecologic oncology, two general surgeons and gynaecologists undergoing training under their leadership. The steady improvement in optimal cytoreduction reflects improvement in the surgical expertise that occurred over time when the unit functioned as a dedicated gynaecologic oncologic unit. This could be partly due to the members of the team becoming more attuned to complicated procedures over time and partly due to better patient selection (improved imaging techniques and use of laparoscopy in selecting cases for primary surgery).

The critical information required to audit a surgical unit is to be aware of the increase in the number of complex surgical procedures undertaken. We used a surgical complexity score that was developed by Giovanni D. Aletti and his team to assess the extent of surgery [10]. The categorisation of surgeries performed over the years into low, intermediate and high scores, based on surgical complexity score gives an objective assessment of the extent of complex surgeries undertaken. The steady increase in the intermediate score category of surgery performed over the years, reflects the ‘maximal surgical effort’ taken by the team with an objective to reach the goal of no residual disease.

In contrast to primary debulking surgery, NACT can be administered before attempting cytoreductive surgery. The efficacy of NACT followed by interval surgery was evident in several studies [5–7, 13, 14, 15]. We follow the policy of selecting patients for primary surgery or NACT based mainly on the performance status and tumour resectability. The bias of onco-surgeon towards primary surgery hinges on the fact that the increased cytoreduction obtained during interval surgery does not translate to improved survival, i.e., clinical response is not equivalent to oncological response. In addition, occurrence of fibrosis after chemotherapy may make complete resection of macroscopic disease difficult. The present study shows survival benefit in the optimal cytoreduction group irrespective of modality of primary treatment.

There are onco-surgeons who believe in primary cytoreduction for all advanced ovarian carcinomas and others who will give NACT for almost all advanced ovarian cancers. We believe that there is a need to balance both schools of thought and a policy should evolve wherein we should be able to carefully select cases for either treatment depending on the merits of each case, expertise available and resectability. We should identify and develop an algorithm wherein suboptimal cytoreductions can be made near zero. Various investigators have proposed CT criteria [16, 17], CT with peritoneography [18], CA125 [19], micro array analysis [20], or molecular markers to predict operability [20]. Laparoscopy has also has been found to be good in predicting operability [21]. A commentary by Vergote *et al *[22] has outlined an algorithm for choosing primary debulking or primary chemotherapy in ovarian cancer patients.

Since 2010, we have been documenting the result of our surgery as either optimal cytoreduction to no visible residual disease or suboptimal cytoreduction to whatever the size of the largest residual disease. We believe this will help in analysing our surgical efforts better. The aim of all surgery should be optimal cytoreduction to no residual disease.

At the beginning of the last decade, platinum containing chemotherapy became recognised as the standard chemotherapy for ovarian carcinoma. In our study, 96% were treated with platinum containing regimes [23, 24]. The use of platinum with taxanes in 80% cases also shows that the advances in the management of ovarian cancer have been translated sufficiently early to the treated population. The study is in alignment with studies which show that survival depends on the optimal cytoreduction obtained rather than on the primary modality of treatment.

## Conclusions

This study analyses how our clinical practice has evolved over the years. As the evidence for improvement in the outcome of advanced EOC hinges on the optimum debulking achieved and the surgical expertise of the team, we need to identify quality tools to objectively quantify and assess the performance of a team. We find that the optimal cytoreduction rate and the surgical complexity scoring of surgical procedures are effective methods in auditing the performance of a gynaecologic oncology surgical team. The primary modality of treatment does not seem to influence outcome.

## Figures and Tables

**Table 1.** Distribution of patient characteristics.

**Total number of patients 198**		
**Age**	**Frequency**	**Percentage**
<35	10	5
35–50	52	26
>50	136	69
**Pathology**		
Serous	158	80
Poorly differentiated	24	12
Endometrioid	6	3
Mucinous	4	2
Clear cell	4	2
Brenner	2	1
**Stage**		
IIIB	6	3
IIIC	170	86
IV	22	11
**Primary treatment**		
Surgery	82	41.4
NACT	116	58.6
**Preoperative evaluation**		
Tumour markers (CA125)	198	100
CT/MRI Scan	180	91
Laparoscopy	12	6
**Chemotherapy**		
Platinum containing	190	96
Platinum + taxane	158	80

**Table 2.** Sites of residual disease in suboptimally debulked patients.

	Extensive peritoneal disease	Small bowel mesentry	Porta hepatis involvment	Unresectable para aortic nodes	Unresectable diaphragmatic disease
Suboptimal primary cytoreduction	6	7	2	4	5
Suboptimal interval cytoreduction	3	7	1	2	1

**Table 3.** Disease status at the end of follow up.

Disease status	Frequency	Percentage
Disease free	74	37%
Recurrent disease	68	35%
Expired due to disease	35	18%
Lost to follow up	21	10%
